# Evolution of the Structural and Magnetic Properties of Bulk Fe_61_Co_10_B_20_W_1_Y_8−X_Pt_x_ Alloys through the Partial Substitution of Pt for Y

**DOI:** 10.3390/ma13214962

**Published:** 2020-11-04

**Authors:** Pawel Pietrusiewicz, Marcin Nabiałek, Bartłomiej Jeż

**Affiliations:** Department of Physics, Faculty of Production Engineering and Materials Technology, Częstochowa University of Technology, Al. ArmiiKrajowej 19, 42-200 Częstochowa, Poland; nmarcell@wp.pl (M.N.); bartek199.91@o2.pl (B.J.)

**Keywords:** rapidly quenched alloys, structural analysis, functional materials, magnetic properties, FePt in the ferro- and paramagnetic states, Curie temperature

## Abstract

This paper presents the results of an investigation into rapidly quenched Fe-based alloys with the chemical formula: Fe_61_Co_10_B_20_W_1_Y_8−x_Pt_x_ (where x = 3, 4, 5). In these alloys, a small quantity of Pt was added, and the Y content was reduced concurrently. Samples of the aforementioned alloys were injection-cast in the form of plates with the dimensions: 0.5 mm × 10 mm × 10 mm. The resulting structure was examined using X-ray diffractometry (XRD), Mössbauer spectroscopy and scanning electron microscopy (SEM). The results of the structural research reveal that, with a small addition of Pt, areas rich in Pt and Y are created—in which Fe-Pt and Pt-Y compounds, with different crystallographic systems, are formed. It has also been shown that an increase in Pt content, at the expense of Y, contributed to the formation of fewer crystalline phases, i.e., it allowed a material with a more homogeneous structure to be obtained. Magnetic properties of the Fe_61_Co_10_B_20_W_1_Y_8−x_Pt_x_ (where x = 3, 4, 5) alloy samples were tested using a vibrating sample magnetometer (VSM). The magnetic properties of the investigated materials revealed that the saturation magnetisation increased with increasing Pt content, at the expense of Y. This effect is due to the occurrence of different proportions of crystalline magnetic phases within the volume of each alloy.

## 1. Introduction

The development of materials, and the assessment of their performance, are related directly to the evolution of humanity. Alongside the cultural changes, groups of specialists have emerged, committed to the investigation of specific materials. One of these groups specialises in the design and modification of engineering materials. It is well known that changes in the conditions used in the production of metallic materials affect the resulting structures, which in turn can affect indirectly the material properties. In 1956, Turnbull and co-workers developed the theory of crystal nucleation and growth in supercooled liquids [1]. The use of this theory cultivated increasing interest amongst materials engineers in the design of more and more new materials featuring a heterogeneous structure. Only four years after the development of Turnbull’s theory, an amorphous material was produced [2,3]. Over the next 30 years, there was a significant increase in the number of scientists and industry representatives, who were interested in a new group of materials resulting from ultrafast cooling processes. Until the end of the 1990s, the melt-spinning method was one of the most popular methods for producing rapidly cooled alloys. Unfortunately, using this method, it was only possible to produce samples in the form of thin ribbons with a thickness of up to 35 µm—which limited significantly the applicability of such materials. It was not until 1989 that A. Inoue [4,5,6] produced rapidly cooled amorphous alloys with thicknesses significantly exceeding 100 µm. Since then, a new era has begun: that of the rapidly cooled materials known as bulk amorphous materials. A. Inoue stated that to produce an amorphous alloy with greater thicknesses, three conditions must be met: (1) the alloy should consist of at least three components; (2) the difference between the atomic radii of individual elements should be at least 12%; and (3) the main components of the alloy should have negative heat of mixing [6]. Generally, the point is that the glass transition should occur under conditions of significant subcooling at less than the equilibrium crystallisation temperature. This transition is associated with a decrease in enthalpy and liquid alloy volume in a continuous manner to limit the mobility of atoms; this is achieved by increasing the viscosity of the liquid during solidification of the alloy. On the basis of massive amorphous alloys, bulk nanocrystalline alloys with even better properties than their amorphous precursors can be produced [7]. Usually, nanocrystalline alloys are obtained by annealing suitable amorphous materials [8,9,10,11,12]. Such annealing can be carried out in several ways: (1) by prolonged annealing, at well below the crystallisation temperature; (2) by annealing briefly, at close to the crystallisation temperature; and (3) by pulse heating, above the crystallisation temperature. Each of these processes requires complex temperature characteristics and additional treatments that are time consuming and increase the cost of the material production process. There is a method that offers potential for obtaining nanocrystalline material as part of the very process of producing rapidly cooled samples. A nanocrystalline structure can be obtained by minimising the solidification time of the alloy or by appropriate selection of its chemical composition. The best, and most repeatable, results are obtained by usinga method in which the correct choice of chemical composition of the alloy is the deciding factor [13,14,15,16,17,18].

Minor changes in the parameters of the production process affect the structure of the rapidly quenched alloys. So far, the effect of changes in the atomic structure of the amorphous alloys on their resulting magnetic and mechanical properties has not been fully explained. Research papers [19,20] have shown that, using techniques such as micro-cantilever bending or the micropillar splitting approach, it can be proven that microscopic changes in the structure influence the mechanical properties of Zr-Ni alloys. The macroscopic properties differ from the properties of the individual regions within the alloy. These regions are characterised by the disparate atomic order (in the amorphous structure, or already within the crystalline structure). Changes in these properties are not linear, and the properties of the microregions are directly connected with the structure. In the case of the bulk alloys, the magnetic properties are the resultant of properties of many different microregions.

Alloys with added Pt make up an interesting group of materials, with appropriate chemical compositions for enabling the production of nanocrystalline samples. For several years, the authors have been involved in the production, and testing of the ferromagnetic properties, of classical, bulk amorphous, and nanocrystalline alloys [21,22,23,24]. As are sult of numerous literature studies, the authors have found that there are many publications on ferromagnetic materials with added Pt, the samples being produced in the form of thin layers or ribbons [25,26,27,28,29,30,31,32,33,34,35]. This is the reason why the current manufacture and investigations of bulk samples of rapidly cooled alloys with added Pt have been undertaken. The research that the authors have carried out, in connection with these samples, will complement the existing knowledge of rapidly cooled ferromagnetic alloys with added platinum.

This paper presents the results of investigations into the structural and magnetic properties of the following rapidly cooled alloys: Fe_61_Co_10_B_20_W_1_Y_8−x_Pt_x_ (where x = 3, 4, 5). The aim of the study was to implement progressively the partial substitution of Pt for Y and to see how this action affects the glass-forming ability and the formation of crystalline phases. The authors wanted to investigate whether a large difference in negative heat of mixing of alloy components with the largest atomic radii affects the formation of the amorphous matrix.

## 2. Materials and Methods

The studied materials, with the chemical compositions of Fe_61_Co_10_B_20_W_1_Y_8−x_Pt_x_ (where x = 3, 4, 5), were made from high-purity elements: Fe = 99.98%, Co = 99.95%, B = 99.5%, Y = 99.9%, W = 99.95%, Pt = 99.99%. The materials were produced in two stages. The process of obtaining ingots involved the melting of the alloy elements in an arc furnace several times, in order to homogenise their structure. After each melting, the ingot was rotated and re-melted. Pure titanium was melted between the ingot melts, in order to absorb residual oxygen in the chamber. The melting process was carried out using a non-fusible tungsten electrode on a water-cooled copper plate. The first solidification process was carried out with a relatively low current flowing through the electrode (180 A). This treatment allowed for the initial solidification of the alloying elements without losing the weight of the charge (in particular boron). The initial ingots, each with an approximate mass of 10 g, were cleaned—both mechanically and also in an ultrasonic cleaner. Then, the ingots were divided into smaller fragments, which subsequently were used to produce rapidly cooled alloy samples in the form of plates with the dimensions: 10 mm × 10 mm × 0.5 mm. The production of the rapidly cooled plates was carried out using a method involving the injection of each respective liquid alloy into a copper mould (i.e., an injection casting method). The charge was placed in a quartz crucible and melted by induction heating. The liquid alloy was injected into a copper mould under argon pressure. In both cases, the working chambers were evacuated of air and then flushed with argon. The crystalline ingots and rapidly cooled plate samples were produced under a protective atmosphere of argon (at a reduced pressure of 700 hPa). The structure of the resulting samples was examined using X-ray diffraction (XRD-Bruker, Billerica, MA, USA) and scanning electron microscopy (SEM-JEOL JSM-6610LV, Akishima, Tokyo). A Bruker D8 Advance diffractometer, working with an X-ray tube with characteristic Cu-K_α_ radiation (1.54 A), was used for the XRD work. The samples were tested over the 2-angle range from 30° to 100°, with a measuring step of 0.02° and an exposure time of 10 s. All of the XRD investigations were conducted on samples that had been pulverised using a low-energy process, in order to obtain information from the entire volume of each material. The pulverisation process was carried out in an agate mortar under a toluene atmosphere.

The microstructure of the alloys was investigated using a POLON Mössbauer spectrometer (POLON, Kraków, Poland) (equipped with a ^57^Co radioactive source of activity 100 mCi and half-life of 270 days). Analysis of the transmission Mössbauer spectra was performed using the NORMOS software (version 3), developed by R. A. Brand [36].

The distribution of experimental spectra into component spectra was carried out, and the distribution of the hyperfine field induction P(B) was determined. Each experimental spectrum was presented as the sum of elemental sextets according to the Hesse–Rübatsch method [37]:

(1) $$T\left(v\right)=\int_{0}^{\infty }P\left(B\right)L_{6}\left(B,v\right)dB$$

where

P(B)—distribution of the hyperfine magnetic field induction;

L_6_ (B, v)—elementary Zeeman sextet;

v—relative speed of source against the absorbent.

On the basis of the distribution of the hyperfine magnetic field induction, the average value of the hyperfine magnetic field induction, B_hf_, was determined. During the fitting process, due to asymmetry in the Mössbauer spectra, a linear relationship between the isomer shift (IS) and the hyperfine field induction (B_hf_) was assumed [36].

(2) $$IS\left(B_{hf}\right)=IS\left(B_{hf}^{0}\right)-\alpha \left(B_{hf}-B_{hf}^{0}\right)$$

where

B^0^_hf_ = the minimum value of the hyperfine field induction,

α = coefficient.

The surface of each sample cross-section was examined using a scanning electron microscope (SEM) with an accelerating voltage of 20 kV. The magnetic properties of each sample were measured using a LakeShore VSM 7307 (Carson, CA, USA) vibrating sample magnetometer for magnetic field strengths up to 2 T. The measurements were carried out in the perpendicular directions and vertical to the plate in order to determine the presence of a favoured axis of magnetisation.

The saturation magnetisation polarisation curves were measured using a Faraday balance (AGH, Kraków, Poland). The measurement was carried out over the range from room temperature to 1100 K. The samples were heated at a rate of 10 K/min. Curie temperature was determined using the relationship:

(3) $$\mu_{0}M\left(T\right)=(\mu_{0}M_{0}){\left(1-\frac{T}{T_{c}}\right)}^{\beta }$$

## 3. Results

**Figure 1:**
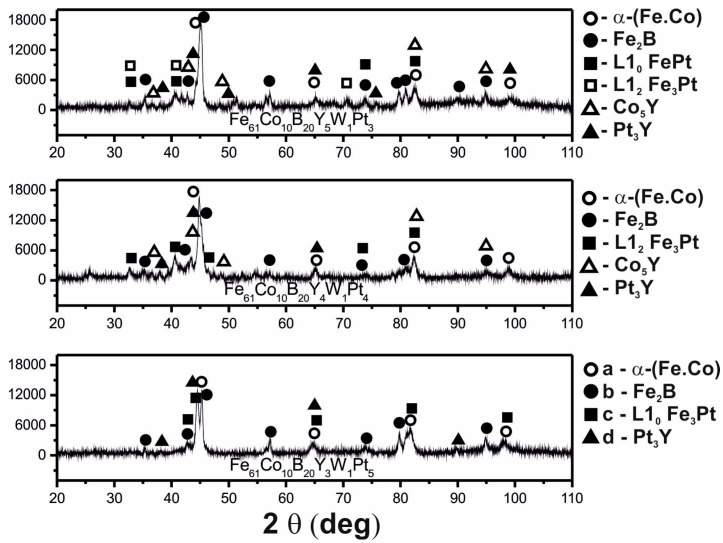
X-ray diffraction patterns for the plate-form samples of the investigated alloys.

In Figure 1, X-ray diffraction images are presented for the alloys Fe_61_Co_10_B_20_W_1_Y_8−x_Pt_x_ (where x = 3, 4, 5). The X-ray diffraction images for all of the tested alloys were found to be similar, and within their volume, there are two types of arrangement of the atoms: short- and long-range. This means that, during the production process, it was possible to “freeze” part of the amorphous matrix structure using the chosen production parameters. Given the same production parameters, changes in the percentages of Y and Pt, within the alloy sample volume, resulted in the creation of different crystallographic systems: α-Fe, Fe_2_B, L1_0_ FePt, L1_2_ FePt_3_, L1_2_ Fe_3_Pt, Co_5_Y, Co, Pt_3_Y, and L1_0_ Fe_3_Pt. The crystalline phases α-Fe, Fe_2_B, and Co_5_Y are well described in the literature [38,39] and do not require further discussion. The FePt-containing crystalline phases with L1_0_ and L1_2_ elemental cells are much more interesting and less well-known. All of the FePt phases have magnetic properties. The L1_0_FePt and L1_2_ Fe_3_Pt phases form crystallographic systems with tetragonal and cubic cells, respectively, and have ferromagnetic properties [40]. The Fe_3_Pt crystalline phase must be separated into two phases with the designations L1_2_ and L1_0_, which have soft and hard magnetic properties, respectively. In contrast, the FePt_3_ phase, designated L1_2_ (primitive cells), has paramagnetic properties [41,42]. Through analysis of the XRD patterns in Figure 1, it was found that the addition of Pt instead of Y stabilises the structure of the tested alloys. As the Pt content increases, the number of crystalline phases present in the alloy volume is reduced. In accordance with analysis of the phase diagrams for FePt [43] and PtY [44], it was found that within the volumes of the alloys, there are substantial heterogeneities in composition, associated with chemical and topological disorder in the arrangement of the atoms. Topological disorder occurs in both crystalline and amorphous materials, and chemical disorder occurs only in crystalline materials.

Generally, crystalline phases in nanocrystalline alloys are obtained as a result of a stringently designed annealing process, carried out for alloys with an amorphous structure. Nanocrystallisation of amorphous alloys can be performed in one or many stages; in long- or short-term processes. Heating, at a strictly defined rate, and annealing of amorphous samples—in order to obtain nanocrystalline grains within the melt volume—is carried out at a strictly defined temperature. All process parameters are determined on the basis of analysis of the thermal history of the alloy, where the crystallisation temperature is key. Fe-Co-B alloys crystallise mainly in a primary manner with secondary crystallisation, where the main crystallisation product is usually α (Fe, Co).

In the tested materials, in addition to the periodic crystalline systems, there is a proportion of residual amorphous matrix. The crystallisation products emerged from an amorphous matrix during the solidification process.

**Figure 2:**
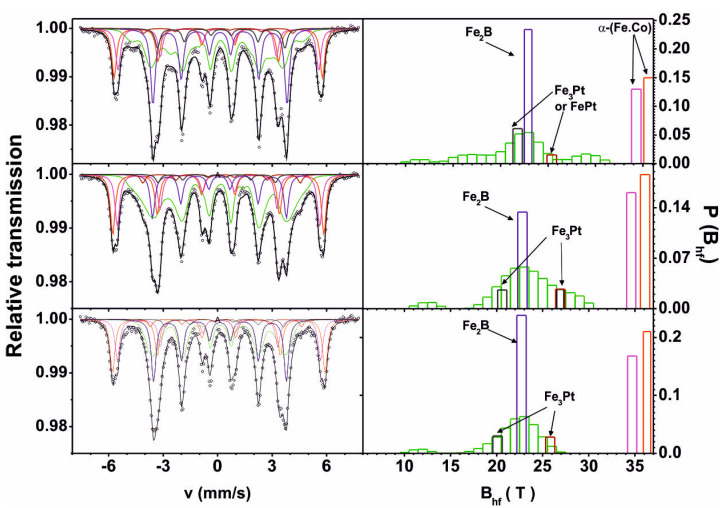
Mössbauer spectra of the samples of the investigated alloys: Fe_61_Co_10_B_20_W_1_Y_8-x_Pt_x_ (where x = 3, 4, 5). Positions of the sub-spectral lines relate to the α-(Fe,Co), α-(Fe,Co)B, Fe_2_B, FePt, and iron-rich Fe_3_Pt.

Figure 2 shows Mössbauer spectra and the hyperfine field distribution on the ^57^Fe nuclei for the investigated alloys.

The transmission Mössbauer spectra, which were obtained for the tested samples, consist of a wide asymmetrical sextet corresponding to the disordered ferromagnetic structure, and five individual sextets corresponding to individual crystalline phases containing Fe. Table 1 presents the results of the analysis of Mossbauer spectra.

Based on the obtained distribution of the hyperfine induction distribution on ^57^Fe nuclei, it can be concluded that the amorphous matrix is heterogeneous. In the case of the Fe_61_Co_10_B_20_W_1_Y_5_Pt_3_ alloy, the distribution of the hyperfine induction on ^57^Fe nuclei indicates several surrounding regions of the central atom. The introduction of more Pt into the alloy—at the expense of Y—results in greater homogenisation of the matrix and separation of the low- and high-field components. Changes in the distribution of Fe atoms in the matrix affect the density of Fe in the crystalline phases formed and their actual share in the volume of the alloy. For the Fe_61_Co_10_B_20_W_1_Y_5_Pt_3_, Fe_61_Co_10_B_20_W_1_Y_4_Pt_4_ and Fe_61_Co_10_B_20_W_1_Y_3_Pt_5_ alloys, the ratios of amorphous matrix to the sum of the crystalline phases are, respectively, 41%/59%, 46.6%/53.4% and 33.6%/66.4%. It is also necessary to consider crystalline phases that do not contain Fe, which cannot be identified using ^57^Co. It should be assumed that the amorphous matrix is rich in Fe and B. For the Fe_61_Co_10_B_20_W_1_Y_3_Pt_5_ alloy, exemplary calculations of the share of crystalline phases in the alloy volume were carried out. In the Mössbauer phenomenon, only Fe atoms are visible, i.e., their percentage assigned to the crystalline phase, described on the basis of the location (in T). For example, a sextet with α-(Fe_70_Co_30_)_100−x_B_x_. The first sextet corresponds to a cell without Boron, the second with one Boron atom in the cell. The first sextet contains 61 × 0.21 = 12.81 Fe atoms and (12.81 × 0.3)/0.7 = 5.49 Co. In the second sextet 61 × 0.16 = 9.76 Fe atoms and (9.76 × 0.3)/0.7 = 4.18 Co and (9.76 + 4.18)/7 = 1.74 B atoms for sextets with B_HF_ 36.3 T and 34.7 T, corresponding to the structure α, (B_HF_ = 36.3 T with intensity 21% and B_HF_ = 34.7 T with intensity 16%), we assign the phase α-(Fe, Co) B, which takes up 34% of the sample volume. The conversion of the composition for this phase gives (Fe_70_Co_30_)_95_B_5_. It follows that (12.81 + 9.76) Fe + (5.49 + 4.18) Co + 1.74B = 34 atoms. This means that almost all of the cobalt is in this phase. We assign a sextet with a field of B_HF_ = 22.7 T and an intensity of 24% to the disordered Fe_2_B phase [45]. It contains 61 × 0.24 = 14.61 Fe atoms and 7.32 Boron atoms. A total of 22 atoms giving 22% content of this phase. The other two sextets are assigned to the Fe_3_Pt phase [46]. The total Fe_3_Pt structure is 61 × (0.03 + 0.03) = 3.66 Fe atoms and 1.22 Pt atoms which gives the content of this phase as 5%. The total proportion of crystalline phases with Fe is 34% + 22% + 5% = 61%. The remaining part of the alloy, i.e., 39%, is an amorphous matrix with folding Fe_51_B_28_(rest)_21_. The calculated percentage of atoms in the identified crystalline phases is given in Table 2.

**Figure 3:**
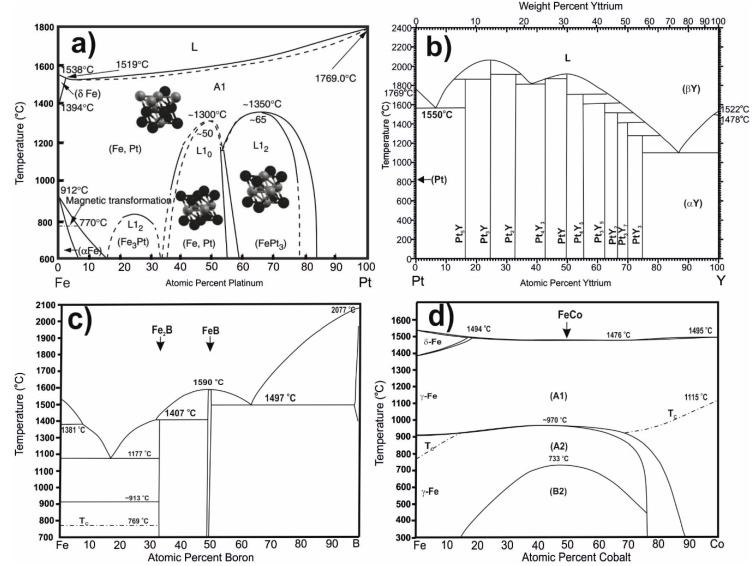
Equilibrium phase diagram and crystallographic structures of (**a**) FePt, (**b**) PtY, (**c**) FeB, and (**d**) FeCo. The figures are based on the cited literature [43,44,47].

As numerous studies have indicated, amorphous materials are characterised by significant fluctuations in composition throughout their volume. This means that completely different crystallographic systems can be formed in different regions of the sample. A deficit of a given ingredient in a certain area becomes a contribution to the formation of specific “crystallisation fronts”, shifting in a favoured direction. This reasoning explains well the presence of crystalline phases present in the tested materials, which are shown in the diagrams of Figure 3 [43,44].

**Figure 4:**
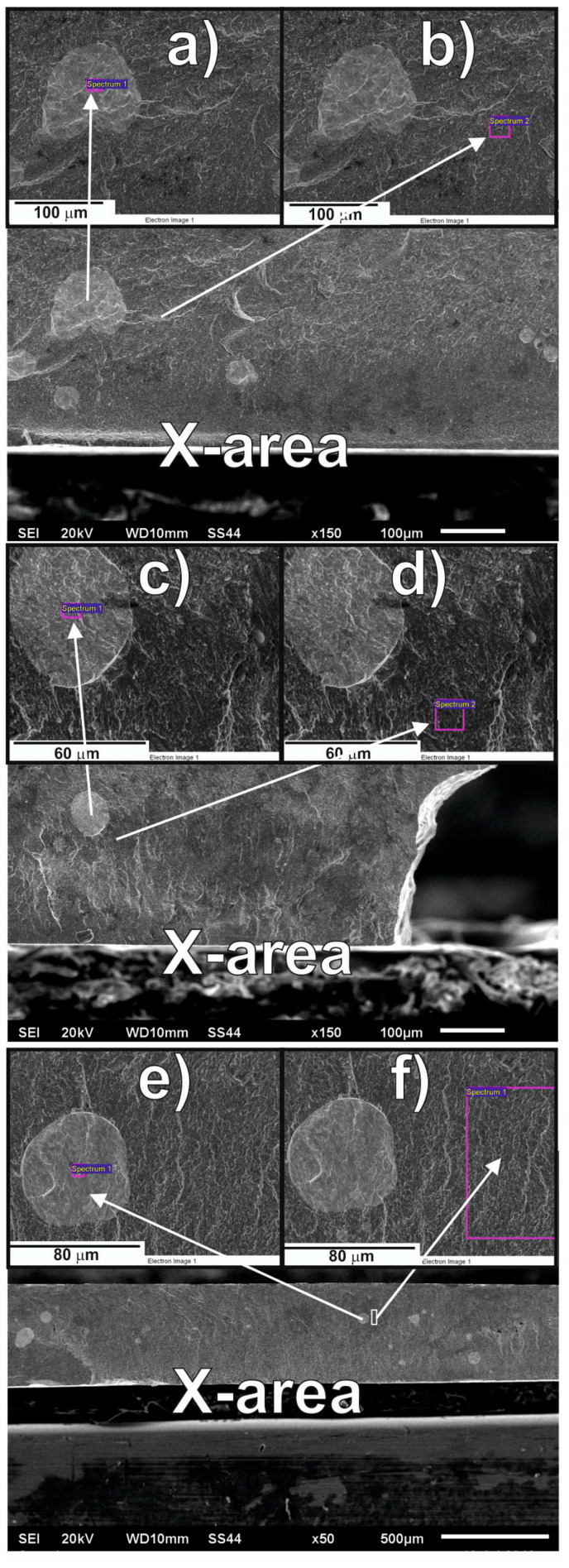
SEM images for the investigated alloys: (**a**,**b**) Fe_61_Co_10_B_20_Y_5_W_1_Pt_3_; (**c**,**d**) Fe_61_Co_10_B_20_Y_4_W_1_Pt_4_; (**e**,**f**) Fe_61_Co_10_B_20_Y_3_W_1_Pt_5_.

Figure 4 shows cross-section images, taken using the SEM, for the investigated samples. Based on analysis of Figure 4, areas marked with an X were selected as sites for detailed investigation. In Figure 4, it can be seen clearly that bright fields of a different chemical composition have formed in the investigated area of the alloy.

**Figure 5:**
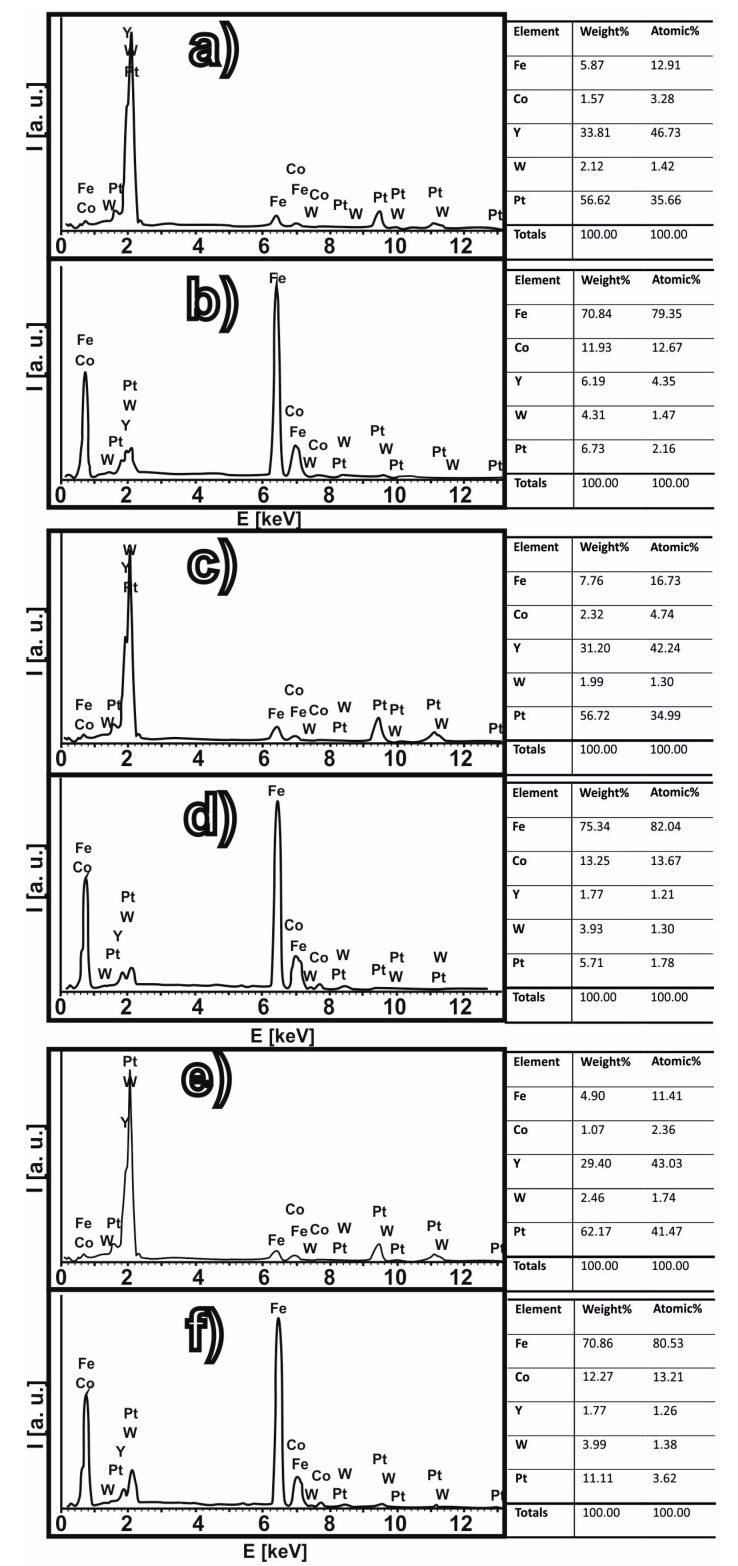
EDS results obtainedfor the investigated alloys, in selected areas of measurement: (**a**,**b**) Fe_61_Co_10_B_20_Y_5_W_1_Pt_3_; (**c**,**d**) Fe_61_Co_10_B_20_Y_4_W_1_Pt_4_; (**e**,**f**) Fe_61_Co_10_B_20_Y_3_W_1_Pt_5_.

Figure 5 shows the results of the EDS (Electron Dispersive Scanning) analysis. The areas in Figure 4 marked as (a), (c), and (e) consist mainly of Y and Pt, with small quantities of the remaining alloy components. The results of the chemical composition analysis indicate that the mass share of Y decreases according to the given formula Fe_61_Co_10_B_20_W_1_Y_8−x_Pt_x_ (where: x = 3, 4, 5); i.e., in the light areas in Figure 4a,c,e. This means that the redistribution of Y is limited the most within the alloy volume. Since the enthalpy of mixing for Y and Pt ΔH^mix^_PtY_ = −83 kJ/mol, the presence of crystal systems containing Pt and Y was to be expected.

Based on EDX analysis, the chemical composition of precipitations on Figure 4a,c,e indicates the presence of not one crystalline phase but several different phases that are rich in Y and Pt: Fe_3_Pt, Pt_3_Y and the disordered arrangement of Y and Pt. The presence of these precipitates should be linked with the interactions between Y and Pt—a very large negative heat of mixing. The melt viscosity increases significantly in the Y and Pt areas. For this reason, these atoms mix with each other and form the areas visible in Figure 4a,c,e. During the rapidly quenched solidification process, most of the Y and Pt atoms (as well as some 236 Fe due to their large number) form crystalline phases while the other atoms (B, Co and the 237 Fe remainder) form an amorphous matrix; i.e., the freezing of the liquid phase structure.

**Figure 6:**
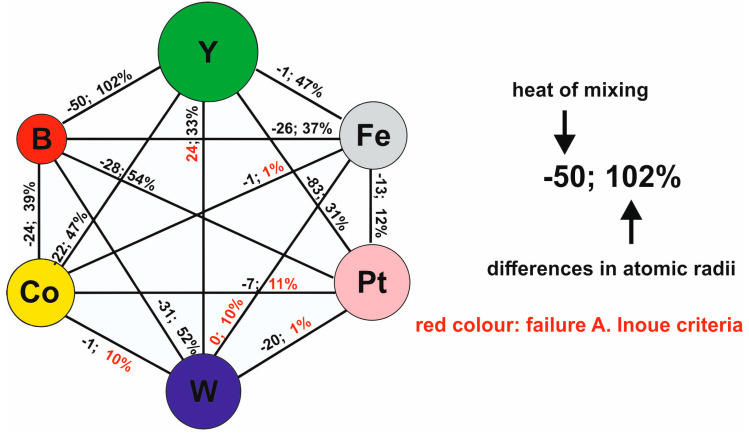
Diagram showing the differences in atomic radii and enthalpy of mixing between the components of the studied materials.

Due to the presence in the alloy of other pairs of elements that have a negative heat of mixing (and notably, couples with Fe), favoured cluster systems form, from which the remaining crystalline phases form during the solidification of the rapidly cooled alloys (Fe-Pt ΔH^mix^_FePt_ = −13 kJ/mol; Fe-B ΔH^mix^_FeB_ = −26 kJ/mol; Co-Y ΔH^mix^_CoY_ = −22 kJ/mol) (Figure 6).

**Figure 7:**
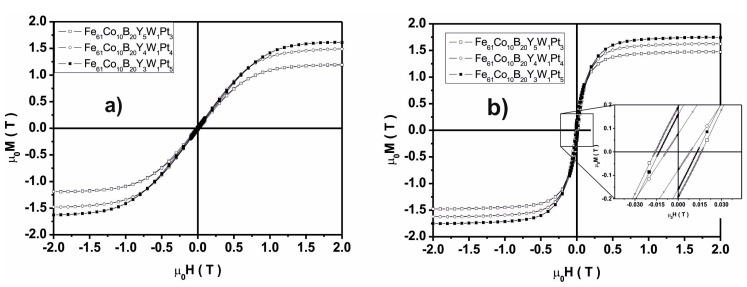
Static hysteresis loops for the investigated samples, measured for the direction of magnetization (**a**) perpendicular, (**b**) parallel to the sample surface.

The static magnetic hysteresis loops, measured for the studied alloys, are shown in Figure 7.

The shapes of the static magnetic hysteresis loops, shown in Figure 7, are similar for the studied alloys. It should be noted that these alloys are ferromagnetic materials. Based on analysis of the shape of the magnetisation process, it can be stated that the investigated alloys are characterised by a relatively large effective anisotropy, as can be seen on the static magnetic hysteresis loops. Two distinct directions of magnetisation could be distinguished, which relates to the production method of the samples (i.e., alignment with the direction of casting). The coercive field value, determined for the studied samples, places them in a group of semi-hard materials. Saturation magnetisation for the tested samples increases linearly with increasing Pt content in the alloy—at the expense of Y. In addition, the increase in magnetisation can be related to the number of crystalline phases present in the sample. Reducing the number of crystalline phases in the alloy volume increases the saturation magnetisation value. An increase in the saturation magnetisation is also connected with an increase in the value of the hyperfine field for α(Fe, Co)B (Table 1). This increase is related to the chemical composition of the phases and distribution of the alloy components. As suggested by the results of the Mössbauer analysis, the α(Fe, Co) phase is doped with B atoms. In addition, the same production process influences the change in the ratio of the Co and Fe atoms, and the change in the value of magnetisation (Table 2).

**Figure 8:**
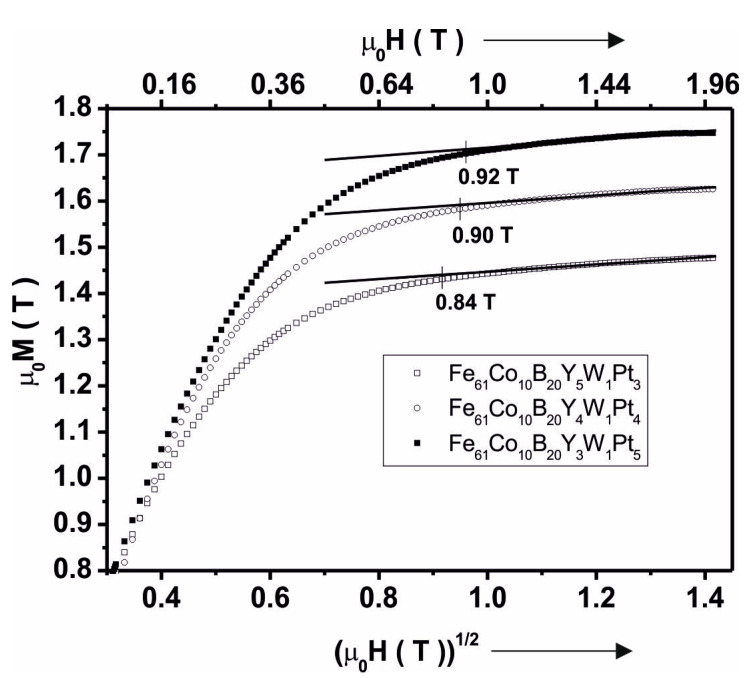
Magnetic saturation (*μ*_0_*M_s_*) of the alloys, in the as-cast state, as a function of (*µ*_0_*H*)^1/2^.

Figure 8 shows primary magnetisation curves, as a function of (μ_0_H)^1/2^, that describe the Holstein–Primakoff para-process [48,49,50].

Using the relationship in Equation (1), the spin–wave stiffness parameter *D_spf_* was calculated.

(4) $$b=3.54g\mu_{0}\mu_{B}{\left(\frac{1}{4\pi D_{spf}}\right)}^{3/2}kT{\left(g\mu_{B}\right)}^{1/2}$$

where

*b*—the slope of the linear fit line *μ*_0_*M_s_((μ*_0_*H)*^1/2^*)*;

*k*—Boltzmann constant;

*μ_B_*—Bohr magneton;

*g*—gyromagnetic factor.

**Figure 9:**
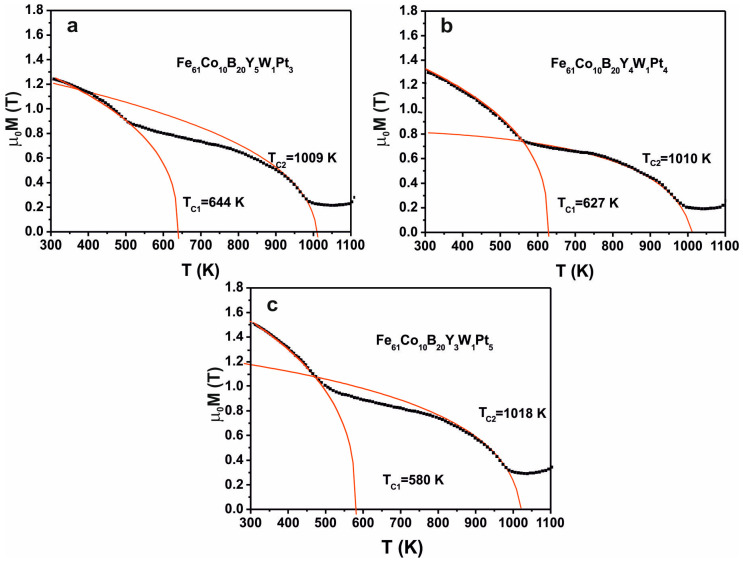
Magnetisation vs. temperature for the as-quenched alloys: (**a**) Fe_61_Co_10_B_20_W_1_Y_5_Pt_3_**, (b**) Fe_61_Co_10_B_20_W_1_ Y_4_Pt_4_, (**c**) Fe_61_Co_10_B_20_W_1_ Y_3_Pt_5_**.**

Changes in the value of the *D_spf_* parameter are related mainly to the content of Y and Pt in the alloy. The large atomic radius of the element Y (180 pm) and its impeded redistribution within the alloy could become a barrier between the magnetic interactions of the pairs of Fe-Fe, Fe-Co, and Co-Co atoms. Table 3 summarizes the results of the magnetic hysteresis loop analysis, obtained using a vibrating sample magnetometer (VSM). Figure 9 shows the curves of magnetic saturation polarisation, as a function of temperature.

The recorded curves show two inflections, related to the transition of ferromagnetic phases to the paramagnetic state. For ferromagnets meeting Heisenberg’s assumptions, it is possible to determine the Curie temperature (T_c_) using the critical parameter β = 0.36 (based on equation 3). The determined T_c_ correspond to the presence of two ferromagnetic phases in the studied temperature range. According to the presented results of structure studies (Mössbauer spectroscopy and X-ray diffraction), the first magnetic phase with a T_c_ of approximately 600 K is the amorphous phase. On the basis of the balance carried out on the basis of Mössbauer spectroscopy, it should be stated that the composition of the amorphous matrix changes, depending on the Pt and Y content in the alloy volume—which is the result of the formation of different crystalline phases in the alloy solidification process. The addition of Pt, at the expense of Y, results in the binding of a quantity of Co in the crystalline phases, in particular in the α(Fe,Co) + B phase. For this reason, there is less Co in the amorphous matrix, which results in a reduction of the Tc of the amorphous matrix from 644 K for the Fe_61_Co_10_B_20_W_1_Y_5_Pt_3_ alloy, 628 K for the Fe_61_Co_10_B_20_W_1_Y_4_Pt_4_ alloy and 580 K for the Fe_61_Co_10_B_20_W_1_Y_3_Pt_5_ alloy. The second determined Curie temperature corresponds to the Fe_2_B crystal phase. According to literature sources, this phase goes paramagnetic at the temperature of 1015 K [26]. For the investigatedsamples, the T_c_ for this phase ranges from 1009 K to 1018 K. Slight differences in the T_c_ value should be related to the fact that the tested alloys were not annealed, and the obtained crystalline phases may contain additional elements that reduce (B) or increase the (Co) T_c_ value. On the basis of X-ray diffraction, four more ferromagnetic phases were identified, which are characterised by the T_c_ value within the selected measuring range: FePt: 750 K, Fe3Pt: 410 K, Co5Y 987 K and α(Fe,Co): 1043. An absence of inflections in the saturation magnetic field curves should be explained by two facts:

- A very small proportion of the phases: FePt, Fe3Pt and Co5Y in the tested alloys;
- Crystallisation of the paramagnetic amorphous phase (which results in an increase in the value of the magnetisation of the sample). Additionally, there is a significant number of Co atoms in the α(Fe, Co) phase, which results in an increase in the Tc value of this phase beyond the measurement range.

## 4. Discussion

Using a method involving the injection of liquid alloy into a copper mould, two-phase samples were produced in the form of plates, containing amorphous matrices and crystalline phases. Based on analysis of their XRD profiles and Mössbauer spectra, it can be concluded that the samples have fine crystal grains with a small proportion of amorphous matrix (in respect of the ratio of intensity to background and peak width). The share of the amorphous matrix in the tested alloys was as follows: 41% for Fe_61_Co_10_B_20_W_1_Y_5_Pt_3_, 46.6% for Fe_61_Co_10_B_20_W_1_Y_4_Pt_4_, and 33.6% for Fe_61_Co_10_B_20_W_1_Y_3_Pt_5_. It was noted that an increase in the Pt content—in place of Y—effects a limitation of the creation of crystalline phases (i.e.,6 to 4 phases for Pt = 3, 4, 5, respectively). It should be mentioned that the production process for each alloy used the same parameters (quantity of load material, injection pressure, chamber pressure and operating current). This means that the only factor affecting the structure of the produced samples was the chemical composition itself. It was assumed that the application of A. Inoue’s criteria would promote the production of samples with a high proportion of amorphous matrix. In the process of solidification of alloys that are produced according to A. Inoue’s criteria, the viscosity increases rapidly, and thisis supposed to reduce the diffusion of component elements. Pt is a component element that accelerates the crystallisation process. This means that the re-distribution of Pt in the alloy is substantial. In the case of the increased viscosity, Pt atoms are re-distributedthroughout the entire volume of an alloy and are pairing with atoms with which they have a negative mixing heat as a balance. For the studied alloys, the energetically preferred option is to combine Y with Pt (ΔH^mix^_PtY_ = −83 kJ/mol), and this is confirmed by the SEM results (Figure 4a,c,e). As shown in the EDS analysis, the quantity of Y in the bright areas decreases with the assumed composition of the alloys, which means that the vast majority of Y connects only to Pt. The remainder of the Y atoms combine into the compound Co_5_Y or occur as a single atom in the volume of the alloys. It is these single Y atoms that can affect the magnetic properties and the saturation magnetisation value. The large atomic radius of Y (180 pm) can be a limitation in the correlation of interchangeable interactions for pairs of magnetic atoms Fe-Fe, Fe-Co, Co-Co, and this is confirmed by the VSM results. The contribution of magnetic phases to the magnetisation process is related to the number of magnetic phases. It can be stated that a larger number of crystalline phases affects the magnetisation process negatively, considering the phases occurring in the investigated samples. Calculationsbased on Equation (1) indicate that it decreases with Pt content.

The analysis of the Mössbauer spectra and hyperfine field distribution shows that the majority of Co is present in the crystalline phase α-(Fe, Co) and a small quantity of it is in the amorphous matrix. With increasing Pt content, at the expense of Y, the hyperfine field value for the phase α-(Fe, Co) increases. This increase is associated with two factors: the quantities of Co and B in this phase. Due to the fact that the tested alloys have not undergone relaxation processes (e.g., annealing), the occurring crystallites are characterised by significant distortion (e.g., expansion). During the rapid-quenching process, the time for diffusion of atoms is much shorter than for gravitational cooling. Hence, during the crystallisation process from the liquid phase, phases doped with otheratomsmay be formed (and are formed). In the case of the tested alloys, the atom absorbed in addition to the phase α-(Fe, Co) is boron. On the basis of the chemical composition balance, based on Mössbauer spectra, the quantities of boron and cobalt in the phase α-(Fe, Co) were estimated. For alloys with a higher Pt content, the hyperfine field values for the phase α-(Fe, Co) reach higher values. This is due to the lower content of B (which reduces the value of the hyperfine field) and the higher value of Co (which increases the value of the hyperfine field). This effect is also visible in the magnetic properties of the tested alloys: The saturation magnetisation value increases with increasing Pt content. This should be associated with a higher hyperfine field value for the phase α-(Fe, Co).

The addition of Pt, at the expense of Y, does not affect significantly the global structure of the tested alloys. Due to the high value of the negative heat of mixing, these two alloying components do not mix well with the other alloying components. In this way, during the solidification process, “islands” are formed, as shown on Figure 4a,c,e. It is worth noting that the chemical compositions of the matrix and the “islands” (determined using EDS) do not differ significantly with the change of Y and Pt content in the tested alloys.

The percentage of the amorphous matrix in the tested alloys is difficult to explain. It does not depend on the chemical composition of the alloy. However, there is a connection between the matrix participation and the magnetic properties—the alloy with the highest proportion of amorphous matrix is characterized by a significantly lower coercive field value.

Thermomagnetic measurements confirmed the presence of the amorphous phase and the Fe_2_B crystalline phase in the tested alloys. Additionally, an increase in Pt content at the expense of Y causes more Co to be bound in the crystalline phases, which results in a lower T_c_ value for the amorphous matrix.

In summary, this study presents the results of an investigation into completely new rapidly cooled alloys, produced in accordance with the established criteria for the preparation of bulk amorphous alloys. The alloys were made in a single-step process, and an analysis of the evolution of the structure, depending on the Pt content has been performed.

## 5. Conclusions

In the investigated alloys, Fe_61_Co_10_B_20_W_1_Y_8−x_Pt_x_ (where x = 3, 4, 5), the number of crystalline phases was found to decrease with increasing proportion of Pt (at the expense of Y). Pt and Y are characterized by high negative heat of mixing (ΔH_mix_PtY = −83 kJ/mol); therefore, there are areas containing mainly Pt-Y in the resulting alloy volume. As the proportion of Pt increases (at the expense of Y content), the magnetisation of the alloy increases; this may be related to the chemical composition of the phase α(Fe, Co). A lower boron content and higher Co content may be responsible for the increase in saturation magnetisation. The effect of amorphous matrix proportion on resulting magnetic properties was identified—the alloy with the highest amorphous phase content has the lowest coercive field value. The increase in Pt content at the expense of Y causes a decrease in T_c_ of the amorphous matrix, which is related to the migration of Co atoms to the crystalline phases during the alloy solidification process.

## Figures and Tables

**Table 1:** The value of the mean hyperfine field using the ^57^Fe nuclei (B_hf_), dispersion of the hyperfine field distributions of the amorphous phase (D_am_), and isomer shift (IS).

Alloys + Crystals		B_hf_ (T)	IS (mm/s)	Total Relative Area	D_am_
Fe_61_Co_10_B_20_W_1_Y_5_Pt_3_	Am	21.18 (20)	0.069	40.99 %	5.88 (17)
α-(Fe,Co)	S1	35.56 (3)	0.038	15.09 %	
α-(Fe,Co)	S2	34.26 (4)	0.044	12.97 %	
Fe2B	S3	22.89 (2)	0.131	23.37 %	
Fe_3_Pt or FePt	S4	25.40 (33)	0.041	1.508 %	
Fe_3_Pt	S5	21.75 (8)	0.196	6.075 %	
Fe_61_Co_10_B_20_W_1_Y_4_Pt_4_	Am	23.02 (12)	0.132	46.61 %	4.60 (14)
α-(Fe,Co)	S1	36.04 (2)	0.389	18.62 %	
α-(Fe,Co)	S2	34.56 (3)	0.521	16.13 %	
Fe2B	S3	22.84 (3)	0.101	13.38 %	
Fe_3_Pt or FePt	S4	26.88 (12)	0.219	2.708 %	
Fe_3_Pt	S5	20.06 (14)	-0.491	2.548 %	
Fe_61_Co_10_B_20_W_1_Y_3_Pt_5_	Am	21.66(19)	0.110	33.61 %	3.292 (30)
α-(Fe,Co)	S1	36.30(2)	0.065	21.00 %	
α-(Fe,Co)	S2	34.68(3)	0.076	15.77 %	
Fe_2_B	S3	22.66 (2)	0.129	23.78 %	
Fe_3_Pt or FePt	S4	25.82 (17)	0.460	2.839 %	
Fe_3_Pt	S5	20.10 (10)	0.369	2.993 %	

**Table 2:** Calculations of the percentage of atoms in the crystalline phases, in relation to the amorphous matrix (per 100 atoms in the alloy), made on the basis of Mössbauer spectra analysis.

Alloys/Crystals Phase	Fe at %	Co at %	B at %	Pt at %
Fe_61_Co_10_B_20_W_1_Y_5_Pt_3_				
α-(Fe,Co)+B	17	7	2	-
Fe_2_B	14	-	7	-
Fe_3_Pt or FePt	5	-	-	2
Sum	36	7	9	2
Fe_61_Co_10_B_20_W_1_Y_4_Pt_4_				
α-(Fe,Co)+B	21	9	2	-
Fe_2_B	8	-	4	-
Fe_3_Pt or FePt	3	-	-	1
Sum	32	9	6	1
Fe_61_Co_10_B_20_W_1_Y_3_Pt_5_				
α-(Fe,Co)+B	23	10	2	-
Fe_2_B	15	-	7	-
Fe_3_Pt or FePt	3	-	-	1
Sum	41	10	9	1

**Table 3:** Data from analysis of the static hysteresis loops (VSM) and application of the Faraday balance (Figure 7 and Figure 9), where *μ*_0_*M_S_*—saturation magnetisation, *H_c_*—coercivity, *D_sp_*—spin-wave stiffness parameter, *T_c_*_1_—Curie temperature for amorphous phase, and *T_c_*_2_—Curie temperature for Fe_2_B phase.

Alloys	*μ*_0_*M_S_* (T)	*H_c_*(A/m)	*D_sp_*(10^−2^meV nm^2^)	*T_c_*_1_ (K)	*T_c_*_2_ (K)	Ref.
Fe_61_Co_10_B_20_**W**_**1**_ **Y**_**5**_**Pt**_**3**_	1.48	**12736**	36	644	1009	[21]
Fe_61_Co_10_B_20_**W**_**1**_ **Y**_**4**_**Pt**_**4**_	1.62	**6608**	35	627	1010	-
Fe_61_Co_10_B_20_**W**_**1**_ **Y**_**3**_**Pt**_**5**_	1.76	**10427**	33	580	1018	-
